# Integrated Multiomics Analysis Sheds Light on the Mechanisms of Color and Fragrance Biosynthesis in Wintersweet Flowers

**DOI:** 10.3390/ijms26041684

**Published:** 2025-02-16

**Authors:** Xuemei Fu, Huabo Wang, Xiang Tao, Yuting Liu, Longqing Chen, Nan Yang

**Affiliations:** Yunnan Province Engineering Research Center for Functional Flower Resources and Industrialization, College of Landscape Architecture and Horticulture Sciences, Southwest Forestry University, Kunming 650224, China; fuxm@swfu.edu.cn (X.F.); 1845205113@swfu.edu.cn (H.W.);

**Keywords:** *Chimonanthus praecox*, color, fragrance, multiomics

## Abstract

Wintersweet (*Chimonanthus praecox*) is known for its flowering in winter and its rich floral aroma; the whole flower is yellow and the inner petals are red. In this study, we chose the wintersweet genotypes HLT040 and HLT015 as the research materials, and studied the co-regulatory mechanism of color and fragrance of wintersweet through metabolomics and transcriptomics. This study found that there were more flavonoids in HLT015, and anthocyanins (cyanidin-3-O-rutinoside and cyanidin-3-O-glucoside) were only present in HLT015, but HLT040 contained more monoterpenes and FVBPs (phenylpropanoid volatile compounds) than HLT015. We constructed putative benzenoids and phenylpropanoid metabolism pathway as well as terpene metabolism pathways. We found some linkages between the different structural genes and metabolites for flower color and fragrance in wintersweet, and screened out 39 TFs that may be related to one or more structural genes in benzenoids and phenylpropanoid or terpene metabolism pathways. In the yeast one-hybrid assay, we found that CpERF7 was able to interact with the promoter of *CpANS1*, while CpbHLH50 and CpMYB21 interacted with the promoter of *CpTPS4*. This study provides a theoretical basis for understanding the co-regulatory mechanism of color and fragrance in wintersweet.

## 1. Introduction

Flower color and fragrance are important ornamental features of ornamental plants. Flower color can attract pollinators and protect floral organs. Plant pigments are classified as chlorophylls, carotenoids, betalains, and flavonoids. As important secondary metabolites, flavonoids belong to the phenylpropanoid class, are widely present in plants, and play crucial roles in plant growth and development [1]. Furthermore, flavonoids have a variety of biological activities, including antioxidant and anti-inflammatory effects, which makes flavonoids a useful raw material for the development of natural health products [2]. Plant fragrances are composed of a diverse array of volatile secondary metabolites, including benzenoids, terpenoids, and aliphatic compounds [3]. Floral fragrances play an important role in plant pollination and signaling [4].

*Chimonanthus praecox* (wintersweet) is a famous traditional ornamental plant in China, which has a long history of cultivation. It has the characteristics of having striking colors, a unique aroma, and winter flowering, and is also a cut flower and bonsai material. The colors of wintersweet flowers are mainly determined by flavonoids; the yellow petals mainly contain flavonoids, such as rutin, kaempferol, and quercetin, while the red petals mainly contain anthocyanins, such as cyanidin-3-O-rutinoside and cyanidin-3-O-glucoside [5,6]. The floral volatile compounds in wintersweet mainly contain terpenes and phenylpropanoids, of which terpenes are dominant [7,8]. The main volatile compounds in wintersweet are β-ocimene, linalool, benzyl acetate, methyl salicylate, etc. [6,9,10], but there are also differences among cultivars; for example, wintersweet genotype SW001 contains trace amounts of linalool and a large amount of ocimene, and H29 contains a large amount of linalool, but less ocimene [11].

In recent years, researchers have sequenced the genome of wintersweet, focusing on flower color and fragrance. Whole genome sequencing and study of flower color were carried out on patens wintersweet, which provided new insights into the molecular mechanism of color development of wintersweet flowers [12]. Through whole genome sequencing and study of the floral aroma regulation mechanism of concolor wintersweet and patens wintersweet, the molecular mechanism of the synthesis of wintersweet fragrance was preliminarily analyzed [13,14]. Regarding the molecular regulation of wintersweet colors, *CpANS1* is a key gene regulating anthocyanin synthesis [5], and some transcription factors have been found to regulate anthocyanin and flavonol synthesis, such as CpMYB1, CpbHLH2, CpMYB2, and CpbHLH1 [12,15,16]. Presently, functional research on the biosynthesis of wintersweet fragrances mainly focuses on structural genes, such as *CpFPPS* [17], *CpGPPS* [18], *CpSAMTs* [13,14,19], and *CpTPSs* [11,13,14,20].

The synthesis of phenylpropanoid volatile compounds and flavonoids comes from the phenylpropanoid metabolism pathway, and the upstream pathways of terpene volatile compounds and carotenoids are the same [3,21]; Thus, the regulatory mechanisms of flower color and fragrance synthesis might have some commonality. Owing to sharing of the same substrate, there is inevitably competition for resources in the synthesis of flower color and fragrance, which is reflected in differences in the structural gene expression of plants, and further manifests in differences in the regulation of structural genes by regulatory factors, such as transcription factors [3,22]. In *Rosa damascena*, there have been studies showing that the overexpression of RdMYB1 increases the expression of *RdPAR*, *RdPAL*, *RdFLS*, *RdRhGT1*, *RdCCD1*, *RdANS*, *RdCER1*, and *RdGGPPS* [23]. PhPH4 regulates the release of phenylpropanoid volatiles, and might affect the accumulation of anthocyanins through its regulatory role in vacuolar acidification [24]. Presently, there are few studies on the correlation between the regulation of flower color and fragrance in wintersweet; one study found that CpbHLH13 not only promotes the synthesis of terpene volatile compounds, but also inhibits that of anthocyanins [25], demonstrating the possibility of researching the co-regulation mechanism of fragrance and flower color in wintersweet.

In this study, through the combined analysis of the metabolome and transcriptome, we analyzed the differences between color and fragrance in two genotypes. We constructed hypothetical color and fragrance metabolic pathways in wintersweet flowers and analyzed the metabolic flux transitions and expression differences in related genes. On this basis, we screened TFs that may co-regulate the synthesis of color and fragrance through co-expression network analysis. Then, we verified the interaction of some transcription factors with the promoters of *CpANS1* and *CpTPS4* using yeast one-hybrid techniques. By integrating the results of multiomics analysis, we explored the relationship between the synthesis of color and fragrance in terms of substances and molecules. The selected structural genes and transcription factors can be used to develop molecular markers related to the color and floral aroma of wintersweet flowers, and then to screen and breed wintersweet cultivars with excellent traits of flower color or fragrance.

## 2. Results

### 2.1. Identification of Differentially Abundant Flavonoids Between HLT040 and HLT015

**Figure 1:**
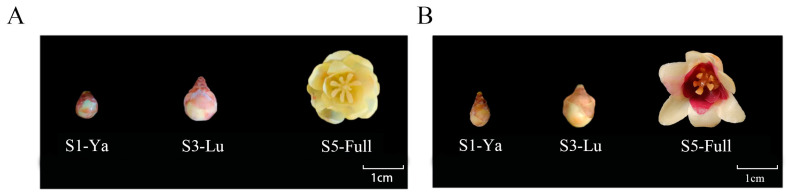
Flower phenotypes of two wintersweet genotypes in three stages. (**A**) Characteristics of HLT040 flower at three growth stages. (**B**) Characteristics of HLT015 flower at three growth stages.

**Figure 2:**
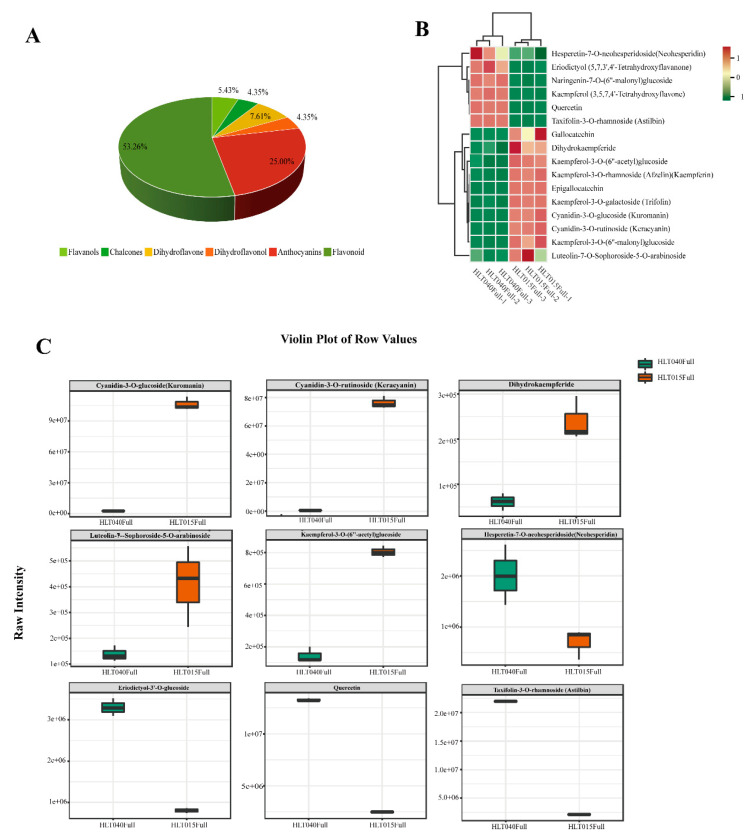
Differential metabolite expression analysis. (**A**) Types and proportions of differential flavonoids between HLT040 and HLT015 flowers. (**B**) Heatmap of 16 differential metabolites with Log2FC > 2 and high levels in HLT040 vs. HLT015. (**C**) Comparison of 9 significantly different flavonoids. Underlying data are from Table S1.

The primary and secondary metabolites of flavonoids in whole wintersweet flower samples in the Full stage were detected using an ultra-performance liquid chromatography–tandem mass spectrometry (UPLC-MS/MS) detection platform, which was also used to comprehensively analyze the flavonoids in wintersweet flowers (Figure 1 and Figure 2). The principal component analysis (PCA) analysis showed significant differences in the substances of the different groups (Figure S1A). In total, 92 differential flavonoids were detected in HLT040 and HLT015 flowers, including 49 flavonoids (53.26%), 23 anthocyanins (25.00%), 7 dihydroflavones (7.61%), 5 flavanols (5.43%), 4 chalcones (4.35%), and 4 dihydroflavonols (4.35%) (Figure 2A, Tables S1 and S2, and Figure S1B). Furthermore, metabolites meeting both |Log2Fold Change (Log2FC)| > 1 and VIP > 1 were considered differential metabolites. The results showed that 51 metabolites were upregulated and 41 were downregulated in the HLT040 and HLT015 comparison (Figure S1D; Table S1). We identified and quantified 17 flavonoids with significant differences between HLT040 and HLT015, of which 11 were more abundant in HLT015. The remaining six metabolites were more abundant in HLT040; this might be responsible for the accumulation of yellow in HLT040 flowers. Importantly, cyanidin-3-O-rutinoside (keracyanin) (7.24 times) and cyanidin-3-O-glucoside (kuromanin) (5.42 times) were the most dominant differentially upregulated metabolites in HLT040 vs. HLT015 (Figure 2C).

### 2.2. Identification of Differentially Abundant Volatiles Between HLT040 and HLT015

**Figure 3:**
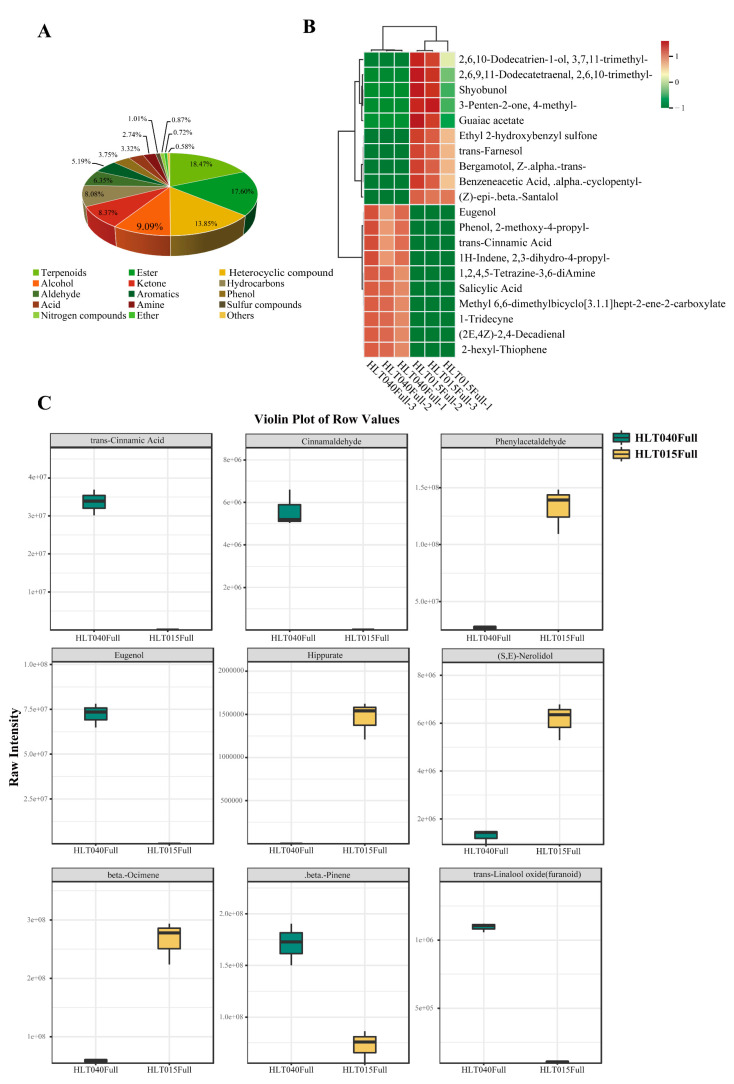
GC-MS was used to analyze volatiles in HLT040 and HLT015. (**A**) Types and proportions of differential volatiles between HLT040 and HLT015 flowers. (**B**) Heatmap analysis of volatile compounds, showing 10 volatiles that were significantly upregulated and 10 volatiles that were significantly downregulated. (**C**) Comparison of 9 terpene and phenylpropanoid volatiles with significant differences. The underlying data are from Table S3.

The floral volatile types and contents in the Full flowering stage of HLT040 and HLT015 were detected via gas chromatography–mass spectrometry (GC-MS). Principal component analysis (PCA) was performed on the metabolome data, and HLT040 and HLT015 were divided into two groups (Figure S2A), which proved that there were obvious differences in the Volatile Organic Compounds (VOCs) of HLT040 and HLT015 at the Full flowering stage. Using a *p*-value ≤ 0.05 and a variable influence on projection (VIP) ≥ 1 as the selection criteria, we identified 693 differential VOCs from the volatile metabolome, of which 370 were higher in HLT040 (Figure S2B,D; Table S3). All differential VOCs could be divided into 15 types, with terpenoids (18.47%), esters (17.60%), and heterocyclic compounds (13.85%) being the most abundant (Figure 3A). A KEGG enrichment analysis of the differential metabolites showed that the differential metabolites were mainly abundant in the monoterpenoid biosynthesis pathway and the phenylpropanoid biosynthesis pathway (Figure S2C; Table S4).

We analyzed the 10 most downregulated and 10 most upregulated compounds in HLT040 vs. HLT015 based on Log2FC values, and found that 6 of the 10 compounds with the most significant upregulation in HLT015 were terpenoids, and 8 of the compounds upregulated in HLT040 were phenylpropanoids (Figure 3B). We compared certain terpene and phenylpropanoid volatile compounds with significant differences in HLT040 and HLT015 flowers, and found that the contents of trans-cinnamic acid and cinnamaldehyde were high and were detected only in HLT040 flowers, and the contents of eugenol (10.09 times), trans-linalool oxide (furanoid) (3.40 times), and beta.-pinene (1.24 times) were higher in HLT040 flowers. Hippurate was detected only in the flowers of HLT015, and the contents of beta.-ocimene (2.18 times), (S,E)-Nerolidol (2.25 times), and phenylacetaldehyde (2.29 times) were higher in the flowers of HLT015 (Figure 3).

### 2.3. Identification of Differentially Expressed Genes (DEGs) in HLT040 and HLT015 Flowers

We performed an RNA-seq analysis of the Ya, Lu, and Full stages of HLT040 and HLT015 flowers, and obtained 127.04 Gb of clean bases (Table S6), with more than 94% of the clean reads mapped to the reference genome (Table S7). Using differential gene screening criteria (|Log2FC| ≥ 1 and *p*-value ≤ 0.05), we identified 16,665 DEGs, including 1330 transcription factors (Tables S8–S11), in HLT040Ya vs. HLT015Ya, HLT040Lu vs. HLT015Lu, and HLT040Full vs. HLT015Full. The DEGs in HLT015Full were abundant in the benzenoid and phenylpropanoid biosynthesis pathway (Figure S3C,D). Combined with the transcriptome and metabolome data analysis, we found 74 DEGs in the benzenoid and phenylpropanoid metabolism pathway and 20 genes in the terpenoid biosynthesis pathway during the three periods of HLT040 and HLT015 (Table S13).

### 2.4. Correlation Between Pigment and Scent Compounds in Wintersweet

Based on the KEGG database and previous studies on the metabolic pathways of color and fragrance [3,22], we constructed the benzenoid and phenylpropanoid metabolism pathway and the terpene metabolism pathway related to wintersweet flower color and fragrance.

(1) The hypothetical benzenoid and phenylpropanoid metabolism pathway

**Figure 4:**
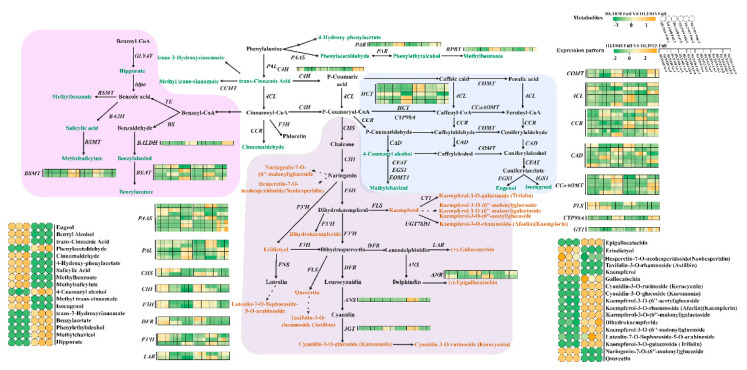
A comprehensive analysis of significantly differential metabolites and biosynthetic genes involved in the benzenoid and phenylpropanoid metabolism pathway in HLT040 and HLT015 flowers. The first color scale in the upper right corner indicates the relative amounts of differential metabolites: green represents a decrease in metabolite content, and yellow represents an increase in metabolite content. The second color mark on the upper right corner indicates the relative expression level of the biosynthetic gene: green represents downregulated expression levels, and yellow represents upregulated gene expression levels. In the pathway, the green font represents volatiles, and the yellow font represents flavonoid compounds. The underlying data are from Tables S5 and S12.

The metabolic pathway can be divided into three branches after producing trans-cinnamic acid and p-coumaroyl-CoA: (1) The benzoic acid synthesis branch: Benzyl alcohol, benzyl acetate, salicylic acid, methyl benzoate, and other FVBPs are synthesized. (2) The eugenol and isoeugenol synthesis branch: Eugenol, isoeugenol, methylchavicol, and other FVBPs are synthesized. (3) The flavonoid synthesis branch: Gallocatechin, keracyanin, kuromanin, and other flavonoids are synthesized (Figure 4).

Regarding floral aroma, 17 FVBPs were found in the phenylpropanoid metabolism pathway. Seven FVBPs (methyl benzoate, benzyl alcohol, methyl salicylate, methyl trans-cinnamate, 4-hydroxy-phenylacetate, salicylic acid, and eugenol) were more abundant in the Full stage of HLT040, and isoeugenol, cinnamaldehyde, and trans-cinnamic acid were only present in the flowers of HLT040. Six FVBPs (phenylethyl alcohol, trans-3-hydroxycinnamate, phenylacetaldehyde, benzyl acetate, methyl chavicol, and 4-coumaryl alcohol) were more abundant in the Full stage of HLT015, and hippurate was only present in the flowers of HLT015 (Figure 4). The floral volatiles of wintersweet are mainly formed and released at the full flowering stage [8], and 27 fragrance-related genes were highly expressed in the hypothetical benzenoid and phenylpropanoid metabolism pathway during the Full stages of HLT040 and HLT015 (Figure 4; Table S12). HLT040 contained higher content of FVBPs than HLT015, including eugenol, trans-cinnamic acid, cinnamaldehyde, methylbenzoate, methylsalicylate, and isoeugenol; these were synthesized by 12 enzymes encoded by significantly upregulated expression genes, including two *CpPAL* genes, one *CpHCT* gene, three *CpCCR* genes, three *CpCAD* genes, one *CpCCoAOMT* gene, and two *CpBSMT* genes. Ten genes (two *CpPAAS* genes, one *CpC4H* gene, two *Cp4CL* genes, two *CpCCR* genes, one *CpCAD* gene, one *CpBALDH* gene, and one *CpBEAT* gene) were highly expressed during the Full flowering period of HLT015, increasing the phenylethylalcohol, phenylacetaldehyde, benzylacetate, methylchavicol, and 4-coumaryl alcohol contents of HLT015 flowers (Figure 4, Table S5).

Regarding flower color, 12 flavonoids with significant differences were screened in the phenylpropanoid metabolism pathway, including 10 flavonoids (gallocatechin, keracyanin, kuromanin, kaempferol-3-O-(6′′-acetyl) glucoside, kaempferin, dihydrokaempferide, kaempferol-3-O-(6′′-malonyl) glucoside, luteolin-7-O-sophoroside-5-O-arabinoside, kaempferol-3-O-galactoside (trifolin), and kaempferol-3-O-(6′′-acetyl) galactoside) which were more abundant in the flowers of HLT015 (Figure 4). The flavonoids of wintersweet flowers are mainly formed in the Ya and Lu stages [6], so we analyzed the expression patterns of flavonoid synthesis-related genes in these two periods. A total of 27 genes were found to be highly expressed in the flavonoid synthesis pathway in the Ya or Lu stages, and 17 genes (62.96%) were significantly higher in the Ya and Lu stages of HLT015 than those of HLT040, including two *CpPAL* genes, four *Cp4CL* genes, one *CpCHS* gene, two *CpF3H* genes, two *CpF3′H* genes, one *CpDFR* gene, one *CpFLS* gene, one *CpANS* gene, one *CpLAR* gene, and two *GT1* genes (Figure 4; Table S12).

Based on the above analysis of color and fragrance compounds and their related genes in the hypothetical benzenoid and phenylpropanoid metabolism pathway, we found that HLT040 had more DEGs involved in forming FVBPs, resulting in the production of a greater amount of FVBPs; conversely, HLT015 had more DEGs involved in forming flavonoids, resulting in the production of more flavonoid compounds.

(2) The hypothetical terpenoid biosynthetic pathway

**Figure 5:**
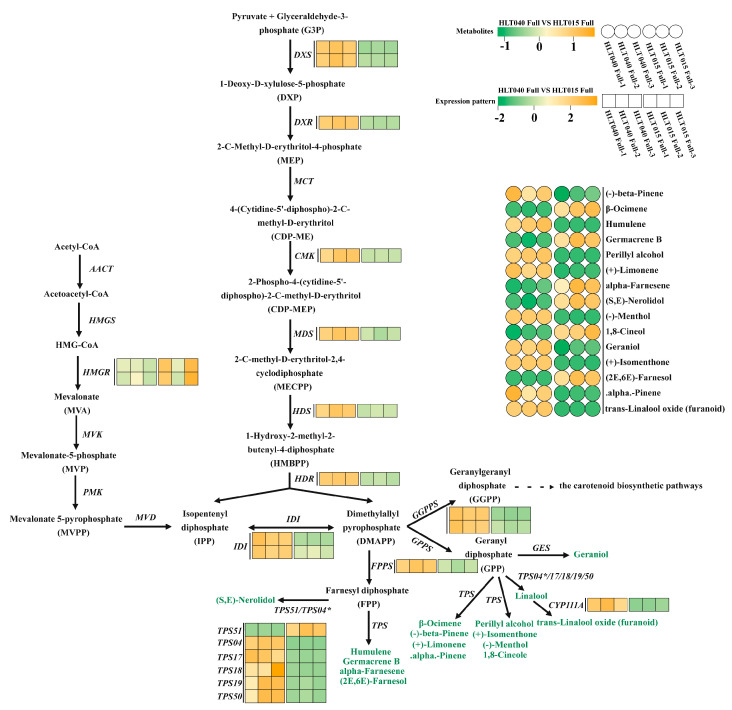
Comprehensive analysis of metabolites and biosynthetic genes with significant differences in terpene synthesis pathways of HLT040 and HLT015 flowers. All legend parameters are shown in Figure 4. The underlying data are from Tables S5 and S12.

In the terpenoid biosynthetic pathway, we found 15 VOCs, of which there was a higher content of 9 (beta-pinene, humulene, germacrene B, perillylalcohol, (+)-limonene, (−)-menthol, (+)-isomenthone, alpha-pinene and furanoid) in HLT040 flowers. Of these, only germacrene B is a sesquiterpene, and the rest are monoterpene compounds, which are synthesized with GPP as a substrate (Figure 5). The genes in the MEP pathway and four *CpTPS* genes (*CpTPS4*, *CpTPS17*, *CpTPS18*, and *CpTPS50*) were significantly upregulated during the full flowering period of HLT040 (Table S12), which led to a significant increase in the monoterpene volatile compound content in HLT040 flowers. In addition, under the regulation of the *CpCYP111A* gene, linalool was extensively oxidized to oxidized linalool (furanoid) in HLT040 flowers. The remaining six terpene volatile compounds were higher in HLT015 flowers, of which three ((2E,6E)-farnesol, (S, E)-nerolidol, alpha-farnesene, and humulene) were sesquiterpenes. Similarly, *CpHMGR* gene and one *CpTPS* gene (*CpTPS51*) in the MVA pathway were significantly upregulated during the Full flowering period of HLT015, significantly increasing amount of the sesquiterpene volatile compounds in its flowers (Figure 5; Table S12).

### 2.5. Screening of Transcription Factors That Regulate Key Genes Involved in Floral Color and Fragrance

We screened the key structural genes regulating flower color and floral aroma in wintersweet based on two metabolic pathways, including the *CpPAAS*, *CpPAL*, *CpC4H, Cp4CL, CpHCT, CpCCR, CpPAR, CpCAD, CpCOMT, CpCCoAOMT, CpBSMT, CpBALDH, CpBEAT, CpBPBT, CpCHS, CpCHI, CpF3H, CpF3′H, CpDFR, CpFLS, CpANS, CpLAR, CpANR, CpGT1*, and *Cp3GT* genes in the benzenoid and phenylpropanoid metabolism pathway, and the *CpDXS, CpHDR, CpDXR, CpGGPPS, CpHDS, CpCMK, CpIDI, CpMDS, CpFPPS, CpHMGR, CpTPS*, and *CpCYP111A* genes in the terpene metabolism pathway (Table S12).

**Figure 6:**
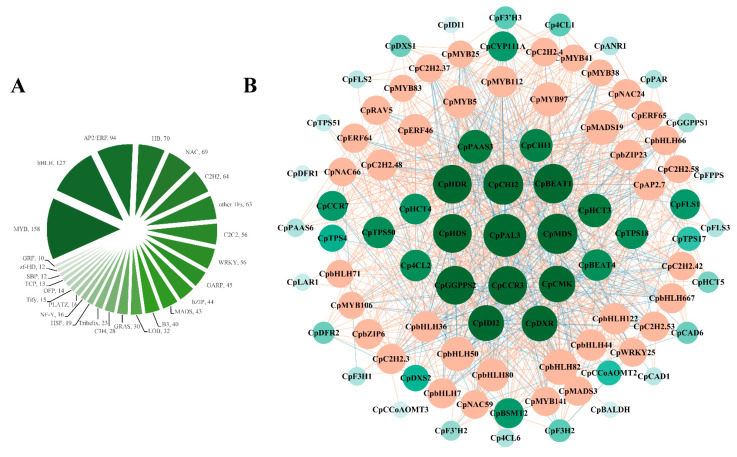
Classification of transcription factors with significantly differentially expressed expressions, and co-expression analysis of structural genes and transcription factors, based on Pearson correlation (PCC). (**A**) Classification and number comparison of significantly differentially expressed transcription factors. (**B**) Green dots represent structural genes; orange dots represent transcription factors. Orange and blue lines represent positive and negative correlations between transcription factors and structural genes, respectively. When PCC > 0.9, structural genes are positively correlated with transcription factor, and when PCC < −0.9, they are negatively correlated. The underlying data are from Tables S15 and S25.

Based on RNA-seq data, we identified 1169 significantly differentially expressed TFs (Table S13). The top 10 significantly differentially expressed transcription factor families were MYB (158 members), bHLH (127 members), AP2/ERF (94 members), HB (70 members), NAC (69 members), C2H2 (64 members), C2C2 (56 members), WRKY (56 members), GARP (45 members), and bZIP (44 members) (Figure 6, Table S15). We conducted co-expression network analysis of eight transcription factor gene families (MYB, bHLH, AP2/ERF, NAC, C2H2, WRKY, bZIP, and MADS gene families, a total of 655 TFs) and 96 structural genes of the phenylpropanoid metabolism pathway and terpene metabolism pathway (Figure S4, Tables S17–S24). In the co-expression network analysis of structural genes, it was found that there were complex expression relationships between structural genes, such as the correlation of the expression patterns of *CpTPS17*, *CpTPS18*, *CpTPS50*, *CpMDS*, *CpBEAT1*, and *CpHCT4* with the expression patterns of more structural genes; there was also a complex expression relationship between flower color-related genes and floral aroma-related genes (Figure S4, Table S16). In the co-expression network analysis of transcription factors and structural genes, we found that 39 transcription factors with expression patterns were highly correlated with the expression of flower color or floral structure genes, mainly transcription factors of the bHLH, MYB, and C2H2 gene families, and the key genes in the co-expression network were *CpHDR, CpCHI2, CpBEAT1, CpHDS, CpPAL3, CpMDS, CpGGPPS, CpCCR3, CpCMK, CpIDI2*, and *CpDXR* (with more than 30 associated TFs) (Figure 6, Table S25).

### 2.6. RT-qPCR Analysis of Key Structural Genes and Candidate TFs

**Figure 7:**
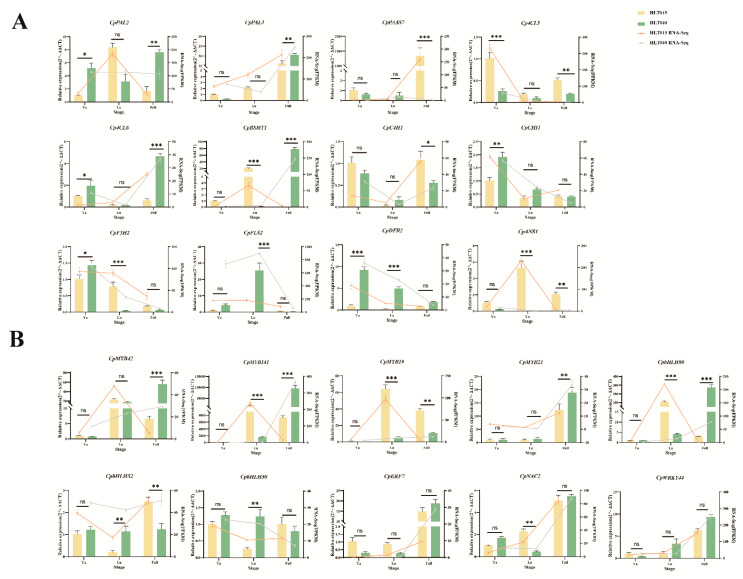
Validation of relative expression levels of key structural genes and TFs involved in floral color and fragrance between HLT040 and HLT015 flowers. (**A**) Relative expression levels of some structural genes in benzenoid and phenylpropanoid metabolism pathway. (**B**) Relative expression levels of some TFs. Polyline represents transcriptome data of gene. ***, extremely significant, *p*-value < 0.001; **, significant, *p*-value < 0.01; *, slightly significant, *p*-value < 0.05; ns, non-significant.

We selected 19 key structural genes and 10 TFs for RT-qPCR analysis (Figure 7 and Figure S5), including 12 biosynthetic genes on the benzenoid and phenylpropanoid metabolism pathway and 7 genes from the terpenoid biosynthetic pathway. These genes play important roles in these pathways, and their expression levels are positively correlated with changes in the corresponding differential metabolites in flower color and fragrance. In different developmental stages of HLT040 vs. HLT015, the trend of RT-qPCR results was basically consistent with that of the RNA-seq data, further demonstrating the reliability of the latter.

### 2.7. Verification of TF Interactions with Flower Scent and Color Promoters

**Figure 8:**
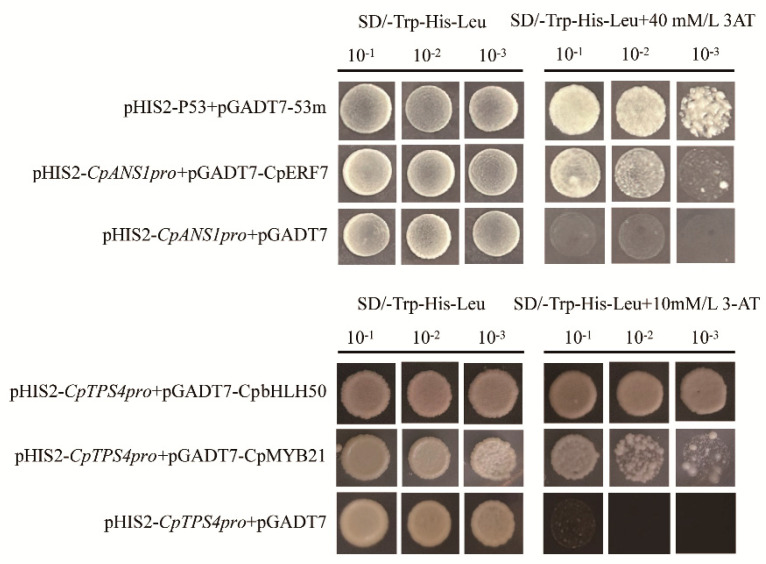
The interactions of transcription factors with *CpANS1* or *CpTPS4* promoters were verified by a yeast one-hybrid assay.

*CpANS1* is a key gene regulating the synthesis of anthocyanins, and *CpTPS4* has been verified to regulate the synthesis of linalool and nerolisol in wintersweet [5,14]. To verify whether the TFs we screened regulate the expression of key structural genes in color or fragrance synthesis pathways, we verified the interactions of some of the TFs with the *CpANS1* or *CpTPS4* promoters using a yeast one-hybrid assay (Figure 8). It was found that yeast could grow normally on SD/-His-Leu-Trp medium with added 3-AT only when the transcription factor was co-transformed with the promoter, indicating that CpERF7 interacted with the promoter of *CpANS1*, whereas CpbHLH50 and CpMYB21 interacted with the promoter of *CpTPS4*.

## 3. Discussion

It is generally accepted that floral terpenoids work together with anthocyanins to attract pollinators, spreaders, and pest enemies, as well as to enhance biotic and abiotic resistance [26,27]. Increasingly, plants show synergistic variations in flower color and fragrance. White and light-colored flowers often contain more volatile compounds than darker-colored flowers, and thus possess a stronger fragrance; such a strategy might ensure that flowers have distinctive characteristics that are more easily recognized by pollinators [28,29,30,31]. This phenomenon also manifests in the lightening of flower color and the extreme reduction in fragrance compounds after a plant has completed pollination, which reduce its attractiveness to pollinators and make it easier for them to identify unpollinated flowers [32]. As a winter bloomer, wintersweet has a greater need for a strong fragrance and bright colors to attract the few pollinators that are active during winter months.

### 3.1. Color and Fragrance Metabolites of Wintersweet Flowers

Previous studies have shown that flavonoids are the main pigments that determine the color of wintersweet flowers [6]. Three flavonols (rutin, kaempferol, and quercetin) have been identified in the flowers of the wintersweet cultivar “Hongyun” and genotype H29, and two anthocyanins, cyanidin-3-O-rutinoside and cyanidin-3-O-glucoside, have been only identified in the red inner tepals [5,12]. In our study, the same three flavonols and two anthocyanins were identified from the metabolome of wintersweet flowers (Figure 2A). Rutin was highly expressed in both HLT040 and HLT015, with non-significant differences in content, whereas the kaempferol and quercetin contents were higher in HLT040 than in HLT015 (Figure 2B; Table S1). Two anthocyanins were screened for high content in HLT015 (Figure 2C). The difference in anthocyanin content was responsible for the red color of the HLT015 inner tepals, which agrees with the results of previous studies [33,34,35]. In addition, we found that hesperetin-7-O-neohesperidoside (neohesperidin), eriodictyol-3′-O-glucoside, and taxifolin-3-O-rhamnoside (astilbin) were present in higher amounts in HLT040. However, previous studies have shown that neohesperidin, eriodictyol, and astilbin are mostly found in citrus, and a large number of studies have focused on their therapeutic role as flavonoids in the treatment of diseases. However, no studies have been conducted on the role of these compounds in plant color development.

Floral volatile compounds appear to be more sensitive to environmental stimuli than anthocyanins, or specific volatile compounds can only be detected at specific developmental stages [36]. The presence of unique floral volatiles in wintersweet flowers is one of its most important ornamental characteristics; it has a complex fragrance composition, owing to the composition of multiple volatile compounds. Previous studies have found that the volatile compounds in wintersweet flowers are mainly formed and released during the full flowering period, and the main volatile compounds in wintersweet flowers are terpenoids and phenylpropanoids, among which the linalool, β-ocimene, benzyl acetate, methyl salicylate, benzyl alcohol, and eugenol contents are high [6,8,37,38]. In our GC-MS analysis of HLT040 and HLT015 flowers, these VOCs were also dominated by terpene volatile compounds and phenylpropanoids (Figure 3A). We found high levels of β-pinene, bornyl acetate, eugenol, and camphene in HLT040, whereas HLT015 flowers had higher levels of β-ocimene, phenylacetaldehyde, and benzyl acetate (Figure 3C; Table S5). We found that the most abundant VOCs in wintersweet flowers with all-yellow petals were benzyl acetate, α-ocimene, and eugenol, while the most abundant VOCs in those with red inner petals were α-ocimene, trans-β-ocimene, and germacrene D [37]. We also found that HLT040 contained more eugenol than HLT015, but more ocimene and germacrene B were detected in HLT015 (Figure 5). We found no significant difference in linalool content in blooming HLT040 and HLT015 flowers, but the difference in oxidized linalool was significant (Figure 3C). Linalool oxide is present in essential oils containing large amounts of linalool [39]. Cis- and trans-pyranoid and furanoid linalool oxides are formed from linalool via the cyclization of 6,7-epoxylinalool [40]. Alternatively, 2,6-dimethyloctan-7-en-2,3,6-triol is generated via the hydroxylation of 6,7-epoxylinalool, followed by acid-catalyzed cyclization, to form linalool oxides [41,42]. The *CpCYP111A* enzyme can catalyze the formation of oxidized linalool from linalool (Figure 5), so the differential expression levels of the *CpCYP111A* gene led to differences in the content of linalool oxide in HLT040 and HLT015.

### 3.2. Hypothetical Color and Fragrance Metabolic Pathways of Wintersweet Flowers

In the benzenoid and phenylpropanoid metabolism pathway, phenylalanine is a common substrate for FVBP and flavonoid formation, and phenylalanine can be directly used as a substrate to produce certain FVBPs. At the same time, after phenylalanine converts into trans-cinnamic acid and p-Coumaroyl-CoA, these can be used as substrates to produce FVBPs with smaller molecular weights before chalcone synthesis; conversely, flavonoids with higher molecular weights can be synthesized with chalcones as substrates [21,43]. According to previous studies on the benzenoid and phenylpropanoid metabolic pathway [3,22], phenylalanine can directly produce phenylacetaldehyde, 4-hydroxy-phenylacetate, methyl benzoate, and other substances through enzyme catalysis in wintersweet’s hypothetical benzenoids and phenylpropanoid metabolic pathway (Figure 4). The metabolic pathway can be divided into three branches after producing trans-cinnamic acid and p-coumaroyl-CoA, which leads to competition for precursors in FVBP and flavonoid formation [44,45]. This competition was evident in HLT015 and HLT040 flowers, especially in the second and third branches; in HLT015 flowers, more flavonoid compounds were formed with p-coumaroyl-CoA as a substrate, but p-coumaroyl-CoA was used as a substrate to produce much more eugenol and isoeugenol in HLT040 (Figure 4). At the same time, because HLT040 produced fewer flavonoids, but the two *CpPAL* genes were highly expressed in the Full stage of HLT040, this resulted in the significant accumulation of the precursor substances cinnamic acid and cinnamaldehyde (Figure 4).

Among the hypothetical terpene synthesis pathways, the terpene volatile compounds in HLT040 and HLT015 flowers were significantly different in the MVA and MEP metabolic pathways (Figure 5). The MVA pathway mainly produced sesquiterpene and triterpenoid compounds [46], and the high expression of the *CpHMGR* and *CpTPS51* in HLT015 led to the synthesis of more sesquiterpene compounds through the MVA pathway, such as (2E, 6E)-farnesol and (S, E)-nerolidol. The MEP pathway mainly produces monoterpene compounds, and is the most important pathway in forming terpene volatile compounds in wintersweet [14]. The DEGs in the MEP pathway were highly expressed in HLT040, resulting in most of the monoterpene compounds being accumulated in HLT040 (Figure 5). In addition, *CpTPS4* is an important gene that regulates linalool synthesis, and it was also highly expressed in HLT040 flowers. The *CpTPS17*, *18*, and *19* genes are thought to be copies of *CpTPS4* [14], and they were also highly expressed in HLT040 flowers, which can lead to more linalool accumulating in the HLT040 flowers. TPS genes are key genes in the synthesis of terpene compound backbones [47], and the high expression of these *CpTPS* genes directly leads to the accumulation of linalool, which was reflected in the accumulation of oxidized linalool in HLT040. Wintersweet contains very few carotenoids [6], which might also be why it produces more terpene volatile compounds.

### 3.3. Co-Regulation of Structural Genes Related to Color and Fragrance Synthesis, Based on Transcription Factors in Wintersweet Flowers

Flower color and fragrance are largely attributable to variations in metabolite fluxes, mainly caused by fluctuations in structural gene expression. The expression of structural genes leads to differences in the content and variety of color and fragrance substances, which, in turn, are regulated by transcription factors. The number of structural genes in a particular plant is much higher than the number of regulatory genes, suggesting that different structural genes might be regulated by the same regulatory genes [48]. Multifunctional transcription factors might regulate color and fragrance compounds [21,30]. Our co-expression network analysis also identified several TFs that might regulate both color and fragrance in wintersweet flowers, including MYB, bHLH, NAC, AP2/ERF, bZIP, C2H2, WRKY, and WADS TFs (Figure 6; Tables S14 and S15).

In wintersweet, some transcription factors have been found to regulate the synthesis of flower color or fragrance, such as CpMYB2 and CpbHLH2 promoting anthocyanin synthesis [15], CpbHLH1 inhibiting anthocyanin synthesis [16], CpMYC2 and CpbHLH13 promoting the synthesis of linalool and β-caryophyllene, respectively [25]. We found that CpERF7 can interact with the promoter of *CpANS1* through Y1H (Figure 8); some studies have found that ERF transcription factors can regulate the synthesis of anthocyanins, such as MdERF78, which can directly act on the promoters of *MdF3H* and *MdANS* to regulate the synthesis of anthocyanins in apples [49]; and transient transformation has shown that PbERF22 can upregulate the expression of *PbCHS*, *PbDFR*, *PbANS*, and *PbUFGT* genes, thereby promoting the synthesis of anthocyanins in pears [50]. We also found that CpbHLH50 and CpMYB21 were able to interact with the promoter of *CpTPS4* (Figure 8). There are many studies on the regulation of TPS genes by bHLH and MYB transcription factors to regulate the synthesis of terpene volatile compounds, such as FhMYB108 [51], CitMYC3 [52], LiMYB1, LiMYB305, and LiMYB330 [53].

Although we found TFs that may regulate the synthesis of anthocyanins and linalool in wintersweet through Y1H experiments, we did not find TFs that co-regulate the synthesis of flower color and floral aroma in wintersweet. Current research has found that CpbHLH13 can both promote the synthesis of β-carboxymethyllene and inhibit the synthesis of anthocyanins [21]; CpbHLH13 has been predicted to interact with CpbHLH112 to regulate the synthesis of fragrance compounds, and CpbHLH13 interacts with CpMYB2 to regulate the synthesis of anthocyanins in wintersweet [54]. The presence of transcription factors with this function has also been demonstrated in some other species, such as AtMYB111, AtMYB12, AtMYB11, PhMYB4, PhODO1, PhEOBI, PhEOBII, AtMYB21, AtMYB24, AtMYB75 (AtPAP1), and AtMYB90, which have been confirmed to regulate the synthesis of flavonols, anthocyanins, and volatile phenylpropanoids [55,56,57,58,59]. AtMYB21 can also form a complex with AtMYC2 to regulate the expression of TPS genes [60]. VvWRKY70 inhibits the biosynthesis of isoprenes and flavonols in grapes [61]. In our study, although we predicted transcription factors that may co-regulate the synthesis of color and fragrance, experiments are needed to prove whether these transcription factors can co-regulate effects on the color and fragrance of wintersweet.

## 4. Materials and Methods

### 4.1. Plant Materials

Two genotypes of wintersweet, HLT040 and HLT015, were obtained from Heilongtan Park in Kunming, Yunnan Province, China. All petals of HLT040 are yellow. The inner petals of HLT015 are red, and the middle petals are yellow (Figure 1). We collected non-petaled flower buds (Ya stage), petaled flower buds (Lu stage), and blooming flowers (Full stage) from HLT040 and HLT015, with three biological replicates in each stage. It takes about 20 days to progress from the Ya stage to the Lu stage, and the petals of the flowers are fully open and the stamens are not closed at the Full stage. All samples were taken in the winter of 2023. All samples were stored in a −80 °C freezer for further transcriptomic and metabolomic analysis.

### 4.2. UPLC-MS/MS Analysis

Flavonoid metabolomic profiling was performed using the flavonoid targeted metabolome method at Metware Biotechnology Co., Ltd. (Wuhan, Hubei, China). A total of 100 milligrams of lyophilized flower powder was dissolved in 1200 µL of extraction solution (70% methanolic water). After multiple vortexing and centrifugation, the supernatant was aspirated and analyzed by UPLC–MS/MS. UPLC–MS/MS analysis of the extracts was carried out according to standard methods and previous studies [62]. Each sample contained three biological replicates. Significantly differential flavonoids between HLT040 and HLT015 flowers were determined by a VIP value ≥ 1 and |Log2FC| ≥ 1.

### 4.3. GC-MS Analysis

The samples were ground into powder with liquid nitrogen, and 0.5 g of each sample was weighed and placed in a 20 mL headspace bottle; then, saturated NaCl solution and 10 μL of 50 μg/mL internal standard solution were added, and each sample was replicated in three replicates. According to the previous research method [63], the volatile metabolome of HLT040 and HLT015 flowers in full flowering was determined. Each sample contained three biological replicates. Significantly differential volatile compounds between HLT040 and HLT015 flowers were determined by a VIP value ≥ 1 and |Log2FC| ≥ 1.

### 4.4. Transcriptome Sequencing and Data Analysis

Total RNA was extracted from flowers of HLT040 and HLT015 at three different stages, RNA was reverse transcribed into cDNA, and the transcriptome data were measured. The transcriptome assay was performed by GenoLab M (GeneMind Co., Ltd, Shenzhen, China), and the analysis method was based on a previous research method [64]. The genome data of wintersweet (https://www.ncbi.nlm.nih.gov/bioproject/PRJNA600650 (accessed on 1 June 2022)) were used as a reference to annotate the transcriptome sequencing data. Each sample contained three biological replicates. We screened and obtained DEGs with a significantly false discovery rate (FDR) < 0.05 and |log2FC| >1.

### 4.5. Co-Expression Network Analysis of Candidate Transcription Factors with Key Structural Genes

Transcriptome data were used to obtain the transcription factors of HLT040 and HLT015 at different stages (Table S13), and the structural genes related to the benzenoid and phenylpropanoid metabolic pathway and terpene metabolism pathway were screened from the transcriptome (Table S12).

Correlation analysis between TFs and structural genes was performed through the online cloud platform (https://cloud.metware.cn). The correlation coefficient between structural genes and TFs that was screened for was the Pearson correlation coefficient (PCC); a correlation was determined with a PCC > 0.8 or <−0.8 and a *p*-value < 0.05. Cytoscape (version 3.10.0) [65] was used to construct co-expression networks of transcription factors and structural genes.

### 4.6. RT-qPCR Analysis

RT-qPCR was carried out for verifying the transcriptome data and analyzing the expression patterns of key genes in wintersweet. We randomly selected some genes from the co-expression network analysis for RT-qPCR analysis. NCBI Primer-BLAST (https://www.ncbi.nlm.nih.gov/tools/primer-blast/ accessed on 9 February 2025) was used to design primers (Table S25). *CpRPL8* was used as an internal reference gene [66]. The RT-qPCR was performed on an eQ9600 (EASTWIN, Suzhou, China) with PerfectStart Green qPCR SuperMix (TransGen Biotech, Beijing, China). There were three sample replicates for each gene, and three technical replicates for each sample. RT-qPCR data were analyzed by Graphpad Pism 9.5.1.733 (https://www.graphpad.com/).

### 4.7. Yeast Y1H

The full-length CD sequences of CpERF7, CpbHLH50, and CpMYB21, and the promoter sequences of *CpANS1* and *CpTPS4*, were mined from the wintersweet genome (https://www.ncbi.nlm.nih.gov/bioproject/PRJNA600650 accessed on 9 February 2025); these sequences were obtained by cloning and sequencing (Tables S26 and S27). Full-length CDs of CpERF7, CpbHLH50, and CpMYB21 were cloned into the pGADT7 vector using EcoRI and BamHI restriction enzymes. Promoter fragments of *CpANS1* and *CpTPS4* were cloned into EcoRI and SacI-digested pHIS2 vectors using EcoRI and SacI restriction enzymes. The pGADT7-CpERF7, pGADT7-CpbHLH50, or pGADT7-CpMYB21 plasmids were co-transformed into yeast strain Y187 using the Frozen-EZ Yeast Transformation II Kit (Zymo Research, Tustin, CA, USA). Transformed yeast cells were selected on DDO (SD/−Leu/−Trp) medium and interactions were tested in TDO (SD/−Leu/−Trp/−His) medium at 30 °C for 3 days.

## Data Availability

The original contributions presented in this study are included in the article/Supplementary Materials. Further inquiries can be directed to the corresponding author(s).

## Supplementary Materials

The supporting information can be downloaded at https://www.mdpi.com/article/10.3390/ijms26041684/s1.
