# Supplementary material for: Integrated Multiomics Analysis Sheds Light on the Mechanisms of Color and Fragrance Biosynthesis in Wintersweet Flowers
Source: Int J Mol Sci. 2025 Feb 16;26(4):1684. doi: 10.3390/ijms26041684 (PMC11855453; doi:10.3390/ijms26041684)
Supplement: Supplementary file 1 [file ijms-26-01684-s001.zip › Supplementary Figure.pdf]

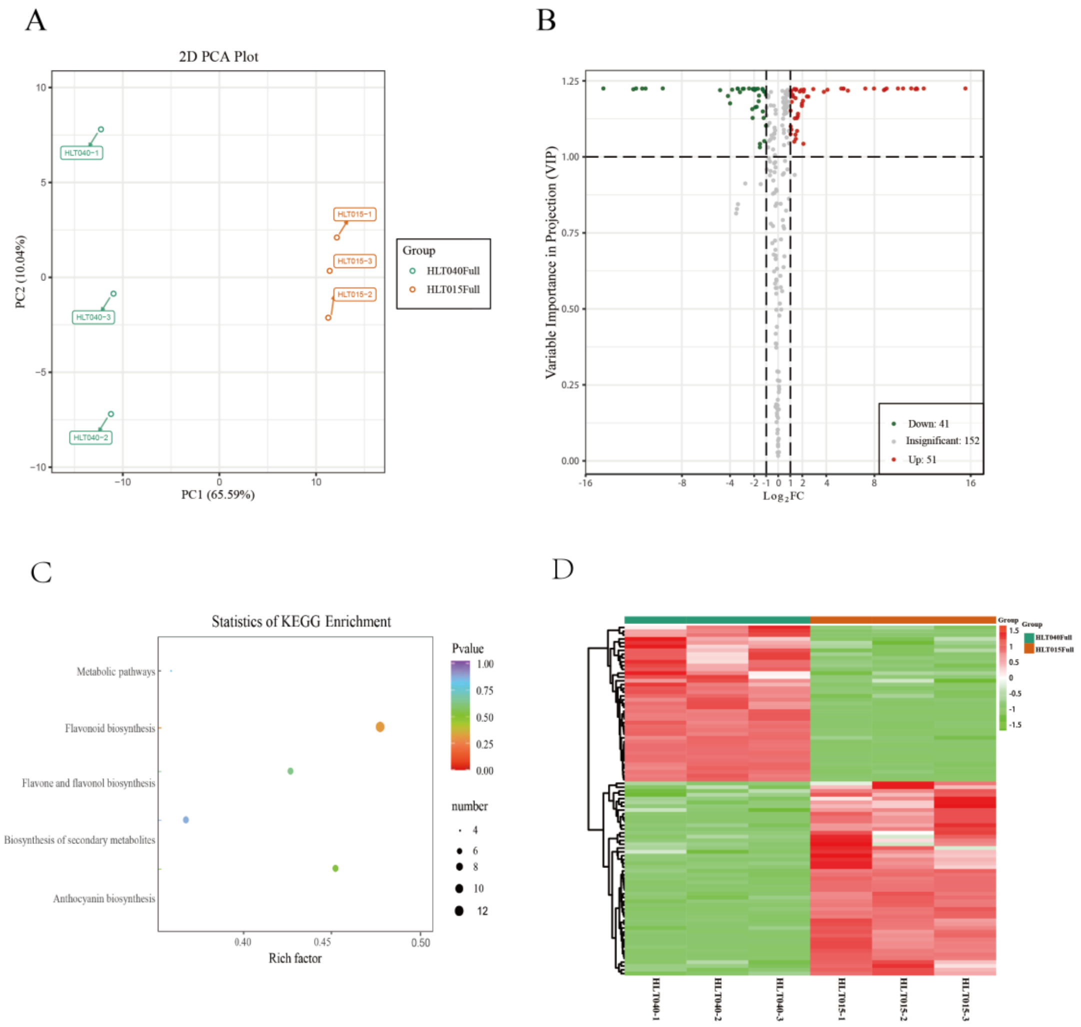

Figure S1. Analysis of flavonoid metabolomes. (**A**) PCA analysis of flavonoids. (**B**) olcanic diagram analysis of flavonoids. (**C**) KEGG enrichment analysis of differential flavonoids in HLT040 and HLT015 flowers. (**D**) Heatmap of differential flavonoids. The underlying data are from Table S1.

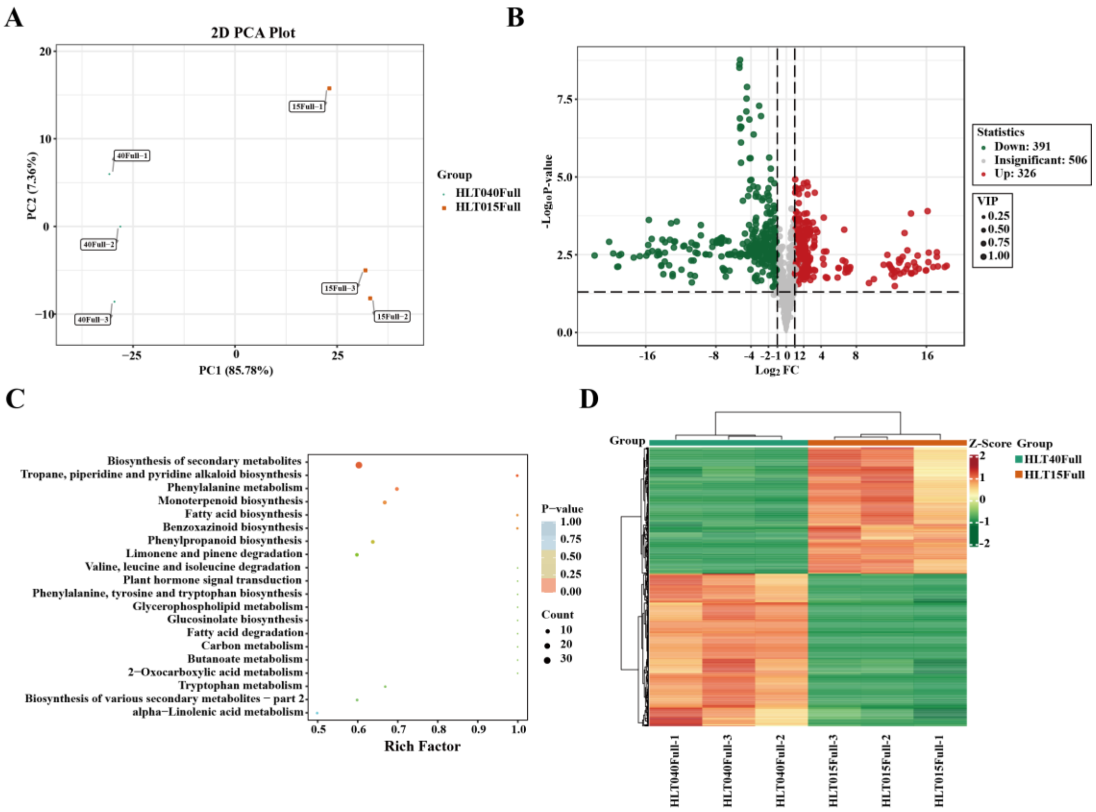

Figure S2. Analysis of volatile metabolomes. **(A)** PCA analysis of volatile metabolites. **(B)** Volcanic diagram analysis of volatile metabolites. **(C)** KEGG pathway analysis of differential volatile metabolites in HLT040 and HLT015 flowers. (**D**) Heatmap of differential volatile metabolites. The underlying data are from Table S3.

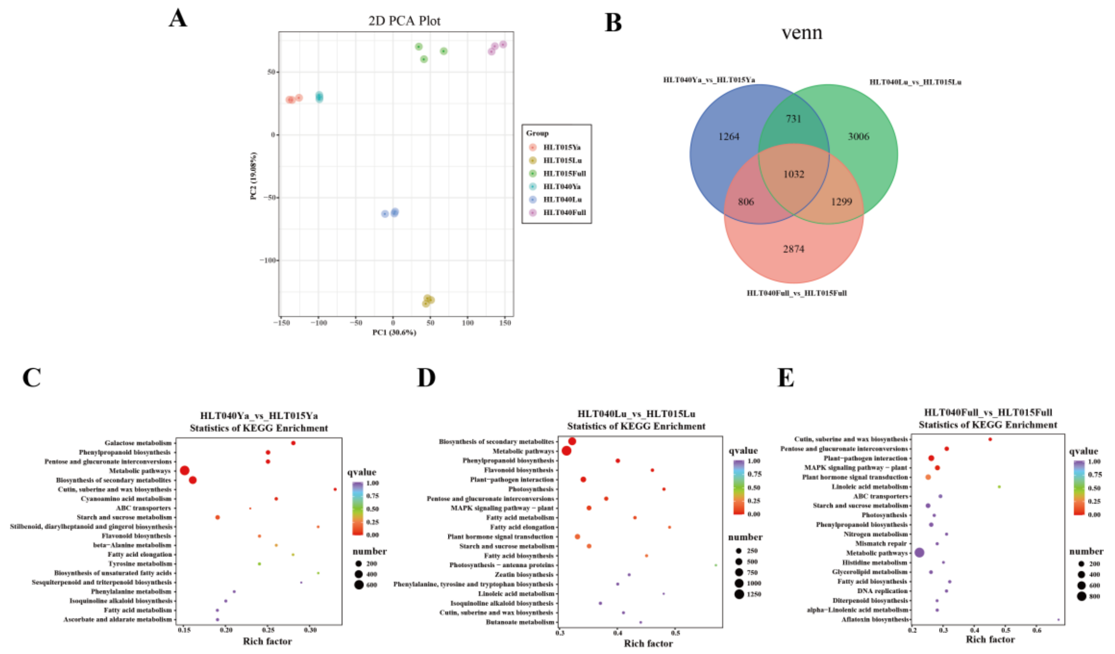

Figure S3. Analysis of the transcriptomes. (A) PCA analysis of the transcriptomes. (B) Venn plots of differentially expressed genes in HLT040 and HLT015 flowers at the three stages. (C) KEGG pathway analysis of differentially expressed genes in HLT040 and HLT015 flowers at the Ya stage. (D) KEGG pathway analysis of differentially expressed genes in HLT040 and HLT015 flowers at the Lu stage. (E) KEGG pathway analysis of differentially expressed genes in HLT040 and HLT015 flowers at the Full stage. The underlying data are from Table S8-S11.

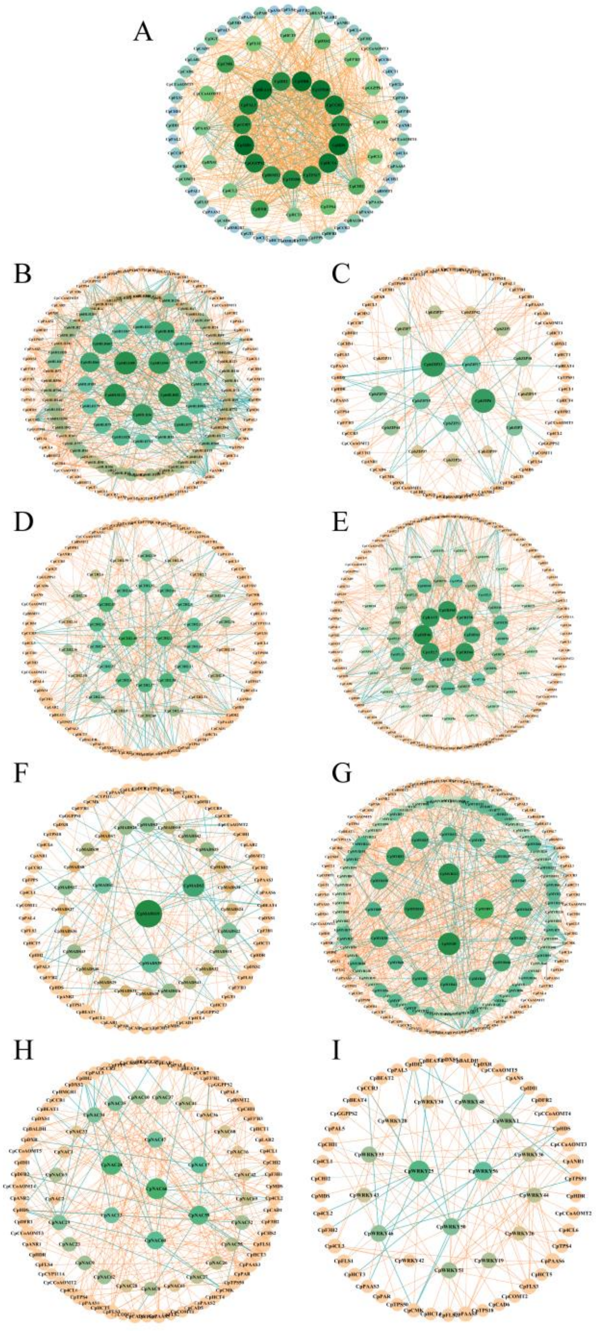

Figure S4. co-expression analysis of structural genes and transcription factors based on Pearson correlation (PCC). (A) co-expression analysis of structural genes. (B-H) The green boxes represent structural genes; the orange boxes represent transcription factors. The orange and blue lines represent positive and negative correlations between transcription factors and structural genes, respectively. When the PCC > 0.9, the structural genes are positively correlated with the transcription factor, and when the PCC < -0.9, they are negatively correlated. The underlying data are from Table S16-S24.

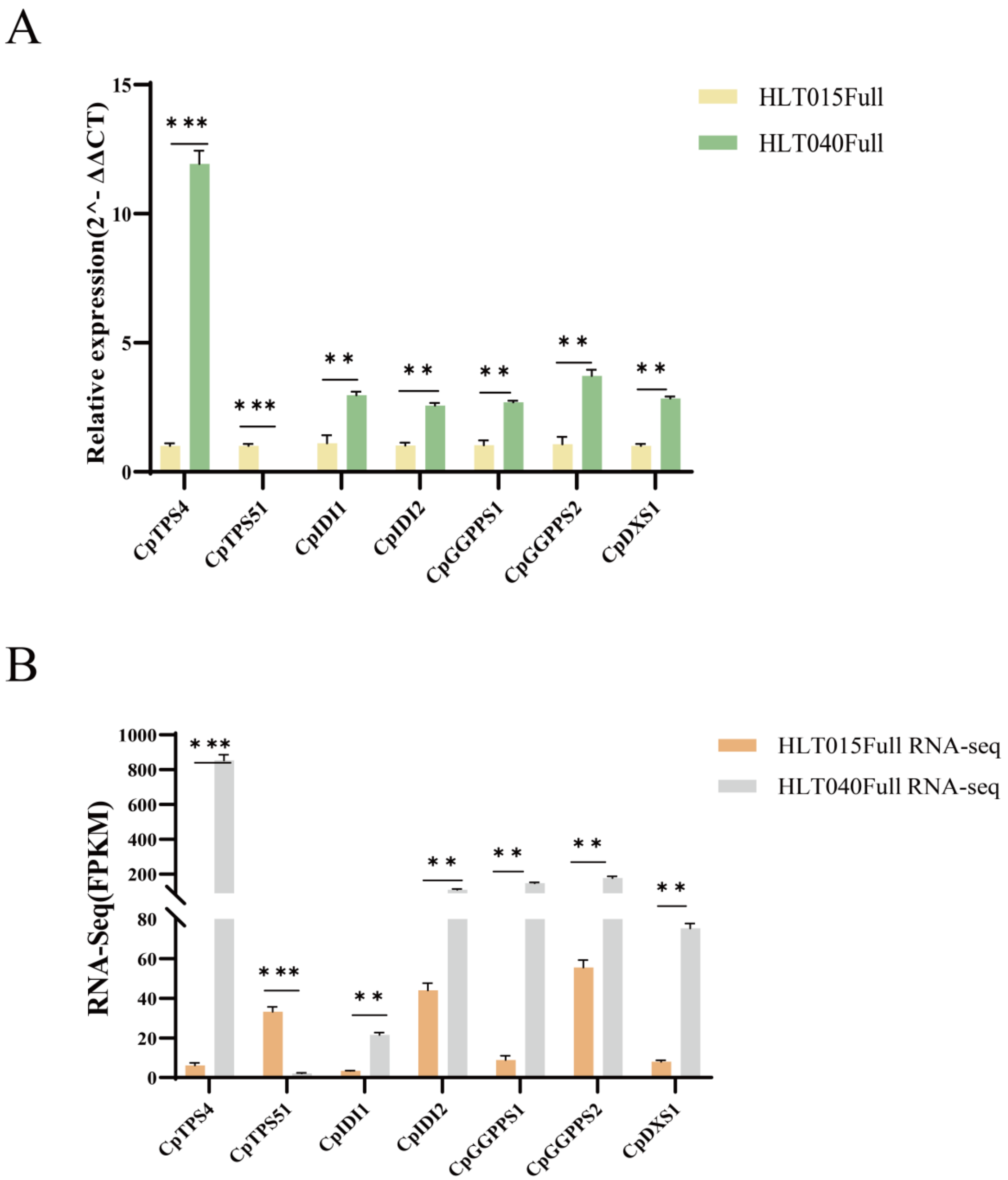

Figure S5. Relative expression levels of some differentially expressed structural genes of HLT040 and HLT015 flowers in the terpene metabolic pathway. (A) Relative expression levels of these genes in RT-qPCR analysis. **(B)** Relative expression levels of these genes in the transcriptomes. ***, extremely significant, P value <0.001; **, Significant, P value < 0.01.
